# Combined analysis of multi-omics reveals the potential mechanism of flower color and aroma formation in *Macadamia integrifolia*

**DOI:** 10.3389/fpls.2022.1095644

**Published:** 2023-02-01

**Authors:** Yonggui Wang, Jing Xia, Zile Wang, Zhiping Ying, Zhi Xiong, Changming Wang, Rui Shi

**Affiliations:** ^1^Key Laboratory for Forest Resources Conservation and Utilization in the Southwest Mountains of China, Ministry of Education, International Ecological Forestry Research Center of Kunming, Southwest Forestry University, Kunming, China; ^2^Yunnan Agricultural University College of Plant Protection, Kunming, China

**Keywords:** *Macadamia integrifolia*, LC-MS/MS, flower color, transcriptome, GC-MS, aroma

## Abstract

**Introduction:**

*Macadamia integrifolia* Maiden & Betche is a domesticated high-value nut crop. The development of nut flower affects the fruit setting rate, yield and quality of nuts. Therefore, in this experiment, two varieties with different flower color, flowering time, flowering quantity and nut yield (single fruit weight) were selected as the research objects.

**Methods:**

Transcriptome (RNA-Seq) and metabolome (LC-MS/MS, GC-MS) analyses were performed to study the regulatory mechanisms of nut flower development, color and aroma.

**Results:**

The results indicated that plant hormone signal transduction, starch sucrose metabolism, phenylpropanoid metabolism, flavonoid biosynthesis, and anthocyanin biosynthesis pathways were related to nut flower development and flower color formation. In the early stage of flowering, most of the differentially expressed genes (DEGs) are involved in the IAA signal transduction pathway, while in the later stage, the brassinolide signal pathway is mainly involved. In starch and sugar metabolism, DEGs are mainly involved in regulating and hydrolyzing stored starch into small molecular sugars in flower tissues. In the phenylpropanoid biosynthesis pathway, DEGs are mainly related to the color and aroma (volatile organic compounds, VOCs) formation of nut flowers. Four color formation metabolites (anthocyanins) in nut flowers were found by LC-MS/MS detection. In addition, the VOCs showed no significant difference between red nut flowers (R) and white nut flowers (W), which was mainly reflected in the aroma formation stage (flowering time). And 12 common differentially accumulation metabolites (DAMs) were detected by GC-MS and LC-MS/MS. At the same time, the DEGs, *AAT*, *LOX* and *PAL* genes, were also identified to regulate key metabolite synthesis during nut flower development. These genes were further verified by qRT-PCR.

**Conclusion:**

Our results provide insights to clarify the molecular mechanism of color and aroma formation during *M. integrifolia* flower development that pave the way for nut quality and yield breeding.

## 1 Introduction

*Macadamia integrifolia* Maiden & Betche is a nut crop with high nutritional value and healthcare functions. Macadamia nuts are rich in unsaturated fatty acids, such as palmitoleic acid (POA). The proportion of unsaturated fatty acids is 85.74%, with oleic acid accounting for 58.60%, arachidonic acid accounting for 15.99%, and POA accounting for 11.15% (Du et al., 2010). It is also rich in protein and vitamins (such as vitamins B1 and B2 and nicotinic acid), which contain about 9% protein and are made up of 18 amino acids. Among the 18 amino acids, glutamic acid and arginine account for a high proportion, including eight essential amino acids, accounting for 28.88% of the total amino acids (Du et al., 2010). Long-term consumption of macadamia nuts can alleviate the occurrence of heart disease (Duxbury, 1995; Garg et al., 2003). Macadamia nut is listed as the most expensive nut because of its higher economic benefits and is considered as the queen of nuts (Stephensonra and Macadamia, 1994). In 2020, China’s *M. integrifolia* planting area of 30 million square meters, ranking first in the world, ranks second in output worldwide. However, the fruit setting rate of nuts is low, generally only 0.1%–0.3% (Urata, 1954; Trueman et al., 1994; Olesen et al., 2011; Mcfadyen et al., 2012), affecting the effective market supply. At present, reports on increasing the yield of *M. integrifolia* are mainly on disease control (Li et al., 2022; Trueman et al., 2022; Qi et al., 2022) and crop cultivation (A'Ida et al., 2021; Russell et al., 2018). In recent years, the research on the *M. integrifolia* breeding system has increased, but there is no report on the molecular mechanism of nut flower development possibly affecting the fruit quality and yield. A low fruit setting rate has become a key factor restricting the yield and industrial development of *M. integrifolia*. There is an urgent need to provide scientific and technical support to solve the problem of low fruit setting rates (Maguire et al., 2004; Herbert et al., 2019; Kämper et al., 2019).

The formation of plants’ flower fragrance volatiles is catalyzed by related enzymes, and there are some differences in the effects of different types of compounds. A previous study found that terpenes and phenylpropanes are the main substances that emit signals for attracting pollinators (Schiestl, 2010). They act as the primary medium between plants and pollinators. Aliphatic compounds, on the other hand, play a defensive role between plants and herbivores. The regulatory genes of the phenylpropanoid pathway are mostly from the MYB family, which is involved in the regulation of the secondary metabolism of flowers and the synthesis of various aroma types (Schiestl, 2010; Xie et al., 2016). *Petunia axillaris* is one of the model plants used to study the flower aroma of plants, and phenylpropanoid compounds are the main source of the flower fragrance of *P. axillaris*. *PhODO1* is the first transcription factor found to promote phenylpropanoid biosynthesis during the odor formation of *P. axillaris* (Xie et al., 2016). The researchers also found that plants usually emit the scent of flowers during periods when pollinators are more active (Hoballah et al., 2005). Like many organisms, there is a corresponding biological clock in plants that enables them to regulate their growth and development rhythms and affect plant metabolites. Many studies have shown that this biological clock plays an important role in the regulation of plant volatiles. However, the effect of flowering time, flower color, and flower fragrance on the yield of macadamia nut has not been reported, and we think this is a subject worthy of study.

In this experiment, two nut varieties with different flower colors, flowering times, flowering quantities, and yield (single fruit weight) were selected as the research subjects. The dynamic changes of the gene expression and metabolite accumulation in nut flowers (red and white nut flowers) at different flowering stages were analyzed using RNA sequencing (RNA-seq), liquid chromatography–tandem mass spectrometry (LC-MS/MS), and gas chromatography–mass spectrometry (GC-MS). This study investigates the molecular mechanism of compound synthesis and related gene regulation in nut flowers during flower development. The results of this study provide a new perspective for the further study of the main metabolites affecting the color and aroma of macadamia nut flowers, which can provide a theoretical reference for the study of nut flower resistance and optimize the quality and yield of nuts. It will also provide a reference for future breeding and cultivation.

## 2 Materials and methods

### 2.1 Plant materials and treatments

The plant materials were collected from three key developmental stages of nut flowers in the “695” (red flower) and “660” (white flower) varieties, which were divided into six groups (i.e., R1, R2, and R3 and W1, W2, and W3). All nut flower samples were collected from Lincang, Yunnan Province, from February to March 2021. The altitude is 850 m (99.259340 E, 24.018357 N). R1 and W1 were at the bud stage (30 days), R2 and W2 were at the half-blooming stage (50 days), and R3 and W3 were at the blooming stage (70 days). The “695” variety (R) has a large number of red flowers that bloom late every year, a medium-sized nut fruit, and is suitable for tropical and subtropical regions (800–1,300 m altitude). The inflorescences of the “660” variety (W) are white, the flowers are short (about 11 cm), and the nuts are small, making them suitable for tropical and subtropical areas (altitudes of 600–1,300 m). All of the samples were whole flowers, which were immediately frozen in liquid nitrogen and stored at −80°C until use. Three biological replicates were utilized at each time point for LC-MS/MS, GC-MS, and RNA-seq analyses. Each repeat includes at least six flowers collected from two to three macadamia nut trees and mixed in equal proportions.

### 2.2 Transcriptome sequencing and DEG analysis

Total RNA was extracted from *M. integrifolia* flowers using the Fast Pure Plant Total RNA Isolation Kit (Vazyme, Nanjing, China). The RNA library was prepared and sequenced in the six *M. integrifolia* flower groups: R1, R2, R3, W1, W2, and W3. The first and second strands were synthesized with random oligonucleotides, SuperScript II, DNA polymerase I, and ribonuclease H, and then the 18 libraries were sequenced on the Illumina sequencing platform (HiSeqTM 2500 or HiSeq X Ten; Illumina, San Diego, CA, USA). After sequencing, the raw reads were filtered and the low-quality reads removed to obtain clean reads. All clean reads were assembled into transcripts by Trinity (Grabherr et al., 2011). The transcripts were hierarchically clustered using the Corset program, and the longest cluster sequence was obtained for subsequent analysis (Davidson and Oshlack, 2014). DESeq2 was used to analyze the input read count data, and the screening thresholds were set as follows: *p*_adj_ < 0.05 and |log2FoldChange| ≥ 1. An independent statistical hypothesis test was carried out on a large number of genes. Finally, the differentially expressed genes (DEGs) in the transcriptome profile were obtained. The function of the unigenes was annotated using Gene Ontology (GO) terms (http://www.geneontology.org) and analyzed with the Blast2GO program. The Blastall software was used to annotate the GO and Kyoto Encyclopedia of Genes and Genomes (KEGG) databases.

### 2.3 GC-MS and volatile organic compound analysis

After vacuum freeze drying, the samples from the different nut flowering periods were ground to powder. For each sample, 500 mg powder was placed in a headspace bottle with saturated NaCl solution and 10 μl (50 μg/ml) internal standard solution. After absorption of the supernatant, the sample was filtered with a microporous membrane (0.22 μm pore size) and then stored in the injection bottle for later GC-MS detection by fully automatic headspace solid-phase microextraction (HS-SPME). In this experiment, the NIST database was used to identify the volatile organic compounds (VOCs) in nut flowers. Multivariate statistical analysis of the VOCs, including principal component analysis (PCA) and partial least squares discriminant analysis (PLS-DA), was performed to reveal the differences in the composition of the VOCs in each comparison group. The variable importance in projection (VIP) value of the first principal component of the PLS-DA model was used and was combined with the *p*-value of the *t*-test to determine the differentially accumulated metabolites (DAMs). The selection criteria were VIP ≥ 1.0, |log2(fold change)| ≥ 1, and *p* < 0.05.

### 2.4 Metabolite profiling by UPLC-MS/MS

The freeze-dried flower samples of R and W at three different developmental stages were weighed and ground with zirconia beads at 30 Hz using a mixer mill (MM 400; Retsch, Haan, Germany) for 1.5 min. Each sample (100 mg) was placed into a centrifuge tube (5 ml), extracted by adding 1,500 μl of 1:1 methanol/water, adsorbed using a CNWBOND Carbon-GCB SPE cartridge (250 mg, 3 ml; ANPEL, Shanghai, China), and then filtered (SCAA-104, 0.22 μm pore size; ANPEL, Shanghai, China). The extract was then fed into the LC–electrospray ionization–MS/MS (LC-ESI-MS/MS) system. The high-performance liquid chromatography (HPLC) system used was the Shimadzu Shim-pack UFLC CBM30A system (www.shimadzu.com.cn/). Based on the method of Liu et al. (2021) with some modifications, the chromatographic column was an ACQUITY UPLC HSS T3 C18 (pore size, 1.8 µm; length, 2.1 mm × 100 mm). For the solvent system, phase A was ultrapure water (adding 0.1% formic acid) and phase B was acetonitrile (adding 0.1% formic acid). The experiment was carried out under an injection volume of 5 μl, a flow rate of 0.4 ml/min, and a column temperature of 40°C. The Thermo QE Focus high-resolution mass spectrometer in information correlation acquisition mode was used to collect high-resolution mass spectrometry data. Three ions with strength greater than 5,000 were selected for each cycle. The mass spectral data were analyzed using MAPS software (Kuhl et al., 2012).

### 2.5 Combined analysis of RNA-seq, LC-MS/MS, and GC-MS

Pearson’s correlation coefficient analysis was conducted between the significant DEGs [with fragments per kilobase per million mapped reads (FPKM) values from the RNA-seq profile] and DAMs (as well as VOCs) in the six nut flower groups, which included certain primary and secondary metabolites. The association between the DEGs, DAMs, and VOCs was analyzed in this study. A correlation coefficient less than 0 represents a negative correlation, while a correlation coefficient greater than 0 denotes a positive correlation. Subsequently, the log2-transformed datasets were loaded in the “cor” package from R software. The top 50 and top 100 DAMs, VOCs, and DEGs were then identified. Finally, the biological significance of modern metabolites was analyzed based on their metabolic pathways and other functions. The DEGs, DAMs, and VOCs were selected using *R*^2^ > 0.9 as the filter criterion, and network diagrams were constructed between the metabolites and the DEGs. Cytoscape software (v3.6) was used for network diagram visualization to represent the relationship between the metabolome and the transcriptome (LC-MS and GC-MS).

### 2.6 Quantitative real-time PCR analysis

Sixteen genes related to metabolite synthesis in flowers were selected for real-time quantitative polymerase chain reaction (qRT-PCR) analysis. These 16 coding genes are involved in nut flower development and color formation and included anthocyanidin 5,3-*O*-glucosyltransferase, flavanone 3-hydroxylase, lipoxygenase, UDP-glycosyltransferase, naringenin, F-box protein, salicylic acid-binding protein, and jasmonic acid-amido synthetase, among others. The primers were designed with Primer 5.0, and their sequences are listed in **Table 1**. RNA was extracted from nut flowers to synthesize the complementary DNA (cDNA). According to the manufacturer’s instructions, qRT-PCR was performed using the ChamQ Universal SYBR q-PCR Master Mix (Vazyme, Nanjing, China). The standard curve was prepared according to the method described by Fan et al. (2017) with *GAPDH* as the internal reference gene. In this study, all genes underwent three biological repeats (with each biological repeat containing three technical repeats). The gene expression level was calculated using the 2^−ΔΔ^*^C^*^t^ method. The normalized relative gene expression level and the FPKM value of the RNA-seq data were calculated as the log2(fold change). R software package 3.1.3 was used to analyze the correlation between the RNA-seq and qRT-PCR data.

**Table 1:** Primers selected for real-time quantitative polymerase chain reaction (qRT-PCR) analysis.

Gene ID		Sequence (5′–3′)	Product size (bp)
Cluster-25626.107700	Forward	TTTGGCACAGTAGGGGCCTT	152
	Reverse	AGTCCCCTCTCCCTTGTACTG	
Cluster-25626.128800	Forward	ATTCACGGGCAAACAACAGG	129
	Reverse	AAACCATCACTCAACGCACA	
Cluster-25626.104601	Forward	GCTTTACTGCCCGAAGGGTT	181
	Reverse	AGAGGGGCCAACTCACCATA	
Cluster-25626.128311	Forward	TGGCAAACGGTCTTCGCTAT	139
	Reverse	TTCCCGACTCAGCACCTCTA	
Cluster-25626.136861	Forward	CAGATGCCGGAGGTCTTACAT	137
	Reverse	ACGGCCTCGATTGCTCTTG	
Cluster-25626.96173	Forward	TCACACCGATCCAGGCACTA	130
	Reverse	GCCAAGGTTGACGACGAAAG	
Cluster-25626.117468	Forward	ACCACAGGAAGAGAAGGAAGC	116
	Reverse	GGAACAAGAAATCCACCCAGC	
Cluster-25626.145312	Forward	GTATCGCCGCCTCTACATGG	168
	Reverse	CTGCCCAAGACCAACCTCTC	
Cluster-25626.70089	Forward	AATCCTAGGTTGCCTCCCGA	200
	Reverse	AGCCTGTACCTGTATAAACCTGC	
Cluster-25626.109979	Forward	GCAATGGATGGTGGTTGTGTG	108
	Reverse	CTGGTTTGGCTGGACACGA	
Cluster-25626.101660	Forward	GTGCAGGTCAAGGACAATGG	200
	Reverse	GTGAGCCACAAGCAAAACCT	
Cluster-25626.109071	Forward	GAGGTCAGAACACAAGGCCA	129
	Reverse	TACAGCAGAATGGTGGCAGG	
Cluster-25626.115681	Forward	CCGGTTATTGAAGCCGCATT	172
	Reverse	CAAAGCCTCCTCGTCAAACC	
Cluster-25626.120871	Forward	CCAGACATTATCACCAACGCAC	181
	Reverse	ACTATTGCTCCTGTTGGGGC	
Cluster-25626.112538	Forward	TTGGGCAAAGGGACAATCCAA	166
	Reverse	AGCAAGGTGTATGGGACCAA	
Cluster-25626.112534	Forward	CTAGATGGCTTCACCGTCGAT	164
	Reverse	GGCTTCAAGGTCCCATCCTC	
*GAPDH*	Forward	CTTCAACATCATCCCTAGCAGC	102
	Reverse	GTGGGAACACGGAAGGACA	

## 3 Results

### 3.1 RNA sequencing, assembly, and quality assessment

**Figure 1:**
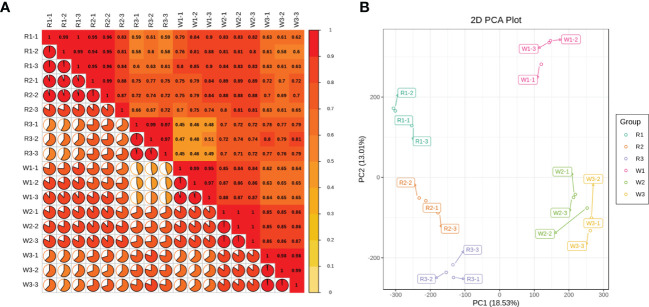
**(A)** Sample-to-sample correlation analysis between each nut flower at different developmental stages. The *numbers in the box* represent the correlation coefficient between each sample; more depth of the *color bar on the right* indicates a higher correlation. The pie chart and the *color* of each sample represent the proportions of the correlation coefficients. **(B)** Principal component analysis (PCA) for each group of nut flowers. *R1*, *R2*, and *R3* represent the three stages of red *Macadamia integrifolia* flowers, while *W1*, *W2*, and *W3* represent the three stages of white *M. integrifolia* flowers. Each color and developmental stage of the nut flower samples had three biological repeats, denoted as *-1*, *-2*, and *-3*.

To study the gene expression and related regulatory pathways of *M. integrifolia* flowers during their critical developmental stages, transcriptome sequencing was performed on 18 samples from two different flower color varieties of *M. integrifolia*. A total of 124.33 Gb of clean data was obtained through sequencing quality control, and the clean data of each sample reached 6 Gb. The percentage of the Q30 bases was >92% (**Table 2**). This result indicates that the assembled genome had high accuracy, contiguity, and completeness. The longest cluster sequence obtained by Corset hierarchical clustering and the spliced unigene N50 was 1,476 bp (**Table 2**). Before the follow-up analysis, we first evaluated the correlation between each sample. The results showed that the correlation of each repetition in nut flowers was greater than 0.9 (**Figure 1A**). As expected, the replicates of each nut flower pattern were clustered together, indicating small variations among replicates.

**Table 2:** Genome sequencing, assembly, and quality assessment in nut flowers.

Type	Number	Mean length	N50	N90	Total bases
Transcript	373,304	854	1,363	347	318,627,966
Unigene	287,486	1,027	1,476	471	295,315,014

The PCA showed that the first principal component could explain 29.89% of the total variance and distinguished the samples based on R (R1, R1, and R3) and W (W1, W2, and W3). The second principal component (PC2) explained 15.04% of the total variance and separated the different flowering stages (**Figure 1B**). The results suggested that there are significant differences among the different nut varieties (**Figure 1**). This may be due to the difference in the genetic background of each variety, or could be caused by the difference in the sampling period. In addition, the different flowering stages of the same nut variety were significantly separated on PC2, and the differences increased with flower development (**Figure 1B**). Subsequently, function annotation was performed on these unigenes, which used the NR, Swiss-Prot, KEGG, GO, and PFAM databases. The annotated unigenes (a total of 287,486) were analyzed for significant enrichment in the different comparison groups.

### 3.2 Analysis of the DEGs in *M. integrifolia* flowers at different stages

DESeq2 was used to analyze the DEGs from each nut flower group at different developmental stages. The screening criteria for DEGs were |log2FoldChange| ≥1 and false discovery rate (FDR) <0.05. Under these conditions, the results of the DEGs in each group are shown in **Supplementary Figure S1**, which quantified the correlation between the PCA and the inter-group samples. The results indicated that regardless of the R or W varieties, the first and third flower development stages had the most number of DEGs (**Supplementary Figure S1**). In the R variety, the numbers of DEGs in the R2 *vs*. R1 and R2 *vs*. R3 comparison groups were comparable. In the W variety, this change seemed to be concentrated in the early stage of flower development (W1 *vs*. W2). Therefore, we next conducted an in-depth analysis of these DEGs.

GO functional analysis was conducted in the R1 *vs.* R2 and R2 *vs.* R3 groups. The 50 significantly enriched terms were identified in the flowers of the two nut varieties (**Supplementary Figure S2**). The results showed that, compared with the DEGs in the R2 *vs.* R3 group, those in the R1 *vs.* R2 group were specifically enriched in photosynthesis, light harvesting in photosystem I, and energy and primary metabolism that involved DEGs, such as NAD activity, amino acid transport, and fatty acid metabolism (**Supplementary Figure S2A**). However, in the late stage of R flower development (R2 *vs.* R3), the DEGs were significantly enriched in secondary regulatory molecular functional terms such as naringenin-chalcone synthase, quercetin 3-*O*-glucosyltransferase, and quercetin 7-*O*-glucosyltransferase (**Supplementary Figure S2B**). At the same time, we also analyzed the GO enrichment of the DEGs at the different stages of W flower development (**Supplementary Figures S2C, D**), which was similar to R. The findings suggested that, during the early stage of nut flower development, the DEGs were primarily associated with the accumulation of primary substances, such as energy metabolism. However, in the later stage, the DEGs mainly regulated the accumulation of the secondary functional components.

### 3.3 Pathway analysis for nut flower color formation

**Figure 2:**
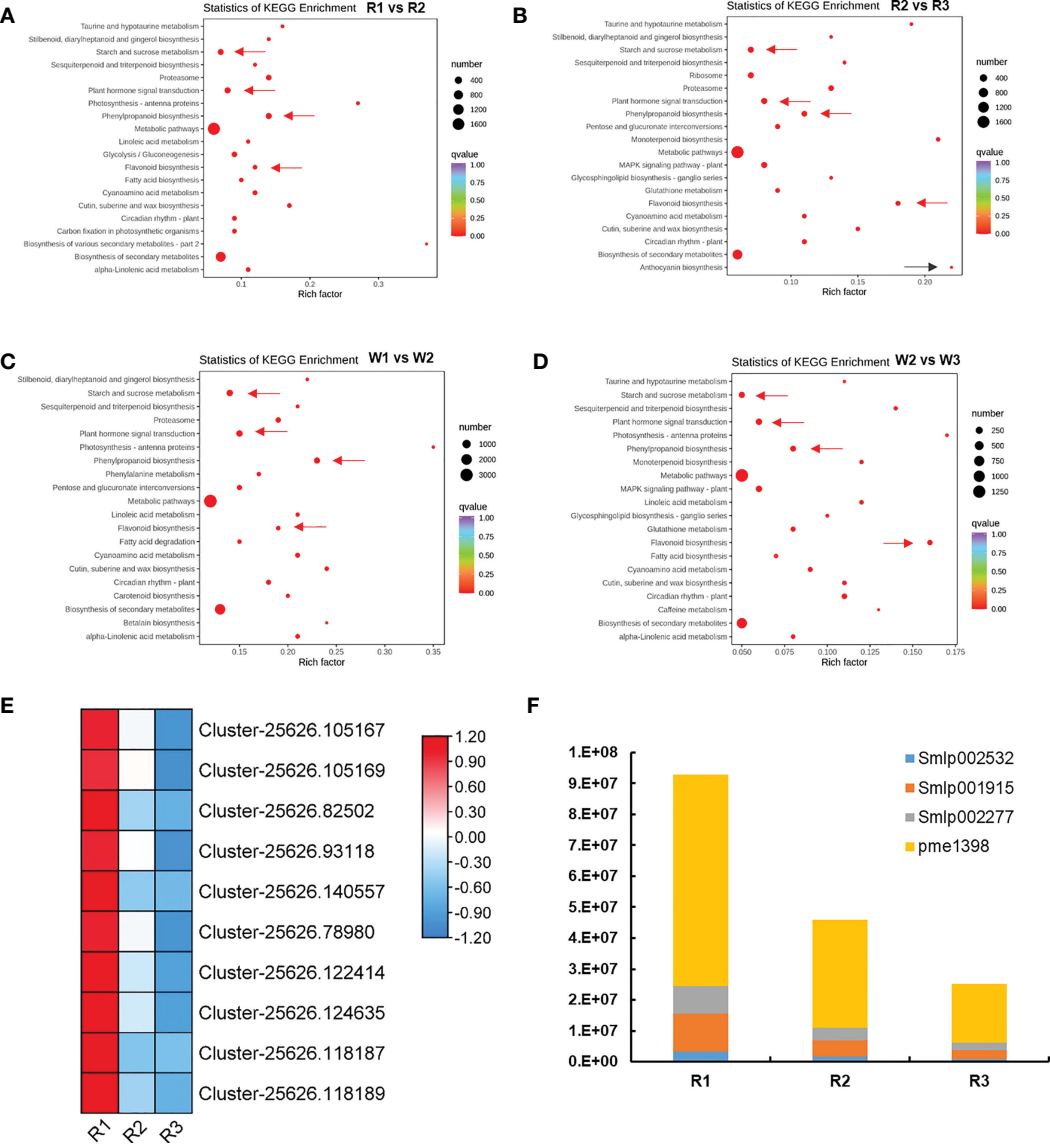
Analysis of the differentially expressed genes (DEGs) and flower color formation during nut flower development (*R* and *W* refer to red and white nut flowers, respectively). **(A, B)** Top 20 of the Kyoto Encyclopedia of Genes and Genomes (KEGG) enrichment of the DEGs in the R1 *vs.* R2 **(A)** and R2 *vs.* R3 **(B)** nut flower comparison groups. **(C, D)** Top 20 of the KEGG enrichment of the DEGs in the W1 *vs.* W2 **(C)** and W2 *vs.* W3 **(D)** nut flower comparison groups. **(E)** Expression changes of the DEGs in the anthocyanin biosynthesis pathway in R nut flowers. **(F)** Changes of the anthocyanin differentially accumulated metabolites (DAMs) in R with the development of nut flowers. The *size of the red dots* in the figure represents the number of enriched DEGs, and the redder the color, the more significant the enrichment. The *arrows next to the red dots* indicate the avenues that this article focused on. *Each square in the heat map* represents a gene, and the *color bars* represent changes in the gene expression, with *red* indicating upregulated gene expression and *blue* downregulated gene expression.

KEGG enrichment analysis was performed in each flowering stage to further examine the color formation and related pathways of *M. integrifolia* flowers. The results showed that plant hormone signal transduction, starch sucrose metabolism, phenylpropane metabolism, and flavonoid biosynthesis pathways were significantly enriched in the different groups of both R and W nut flowers (**Figures 2A–D**). Therefore, we speculated that these pathways are related to *M. integrifolia* flower development and flower color formation. In addition, in the R2 *vs.* R3 comparison group (**Figure 2B**), the DEGs were also significantly enriched in the anthocyanin biosynthesis pathway (ko00942), which may be the main factor for the R (red) flower color formation.

Therefore, we analyzed the changes of the DEGs in the anthocyanin biosynthesis pathway (**Figure 2E**). A total of 10 genes were identified. In the R1 stage, these genes were highly expressed, including anthocyanidin synthase anthocyanidin (*ANS*) (*Cluster-25626.105167*, *Cluster-25626.105169*, *Cluster-25626.82502*, and *Cluster-25626.93118*); anthocyanidin reductase (*ANR*) (*Cluster-25626.140557*, *Cluster-25626.78980*, *Cluster-25626.122414*, and *Cluster-25626. 124635*); and leucoanthocyanidin reductase (*LAR*) (*Cluster-25626.118187* and *Cluster-25626.118189*). The results suggested that these genes are related to the color formation of *M. integrifolia* flowers. In order to accurately identify the key genes related to anthocyanin accumulation and to further elucidate the potential mechanism and chemical basis of the coloration and nutritional quality between R and W flowers, metabolomic analysis was conducted through LC-MS/MS.

Throughout the nut flower opening period, eight key anthocyanins (DAMs) showed a distinctive and specific accumulation pattern, distribution, and fading. These DAMs included cypermethrin-3-arabinoside, deltamethrin-3-arabinoside, petunin-3-*O*-arabinoside, cypermethrin-3-*O*-galactoside, cypermethrin-3-*O*-glucoside, paeoniflorin-3-*O*-glucoside, deltamethrin-3-*O*-galactoside, and trifolin-3-*O*-glucoside (*Additional file 1*). Our analysis revealed that the anthocyanin content was closely related to the color of *M. integrifolia* flower, which is consistent with a previous study (Ma et al., 2022). In the metabolic dynamic changes of the flower color along with flowering, two anthocyanins—delphinidin-3-*O*-rutinoside and cyanidin-3-*O*-rutinoside—played a key role in color formation. In the analysis of the accumulation content of these metabolites, four DAMs were not detected (non-existent) in W, while the accumulation of these DAMs was significantly high in R, with the accumulation content decreasing significantly with the development of flowers (**Figure 2F**). Therefore, based on the phenotypic and transcriptome data of *M. integrifolia* flowers, four possible nut flower chromogenic substances were identified: delphindin-3-*O*-glucoside (Pme1398), petunidin-3-*O*-arabinoside (Smlp002277), delphinidin-3-*O*-arabinoside (Smlp001915), and cyanide-3-*O*-arabinoside (Smlp002532) (**Figure 2F**). The related DEGs for DAM synthesis were verified by qRT-PCR. These DEGs and DAMs were critical for the color formation of *M. integrifolia* flowers.

### 3.4 Effects of the plant hormone pathway on *M. integrifolia* flower development

**Figure 3:**
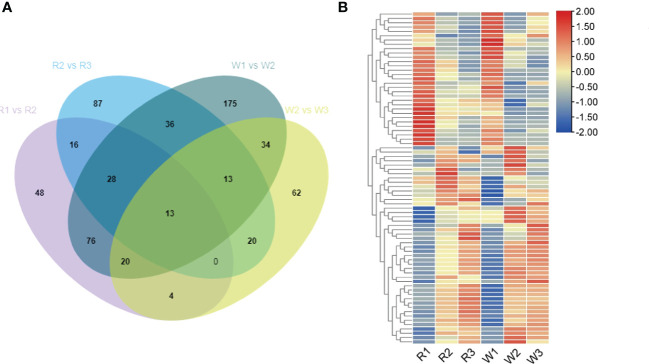
Analysis of the differentially expressed genes (DEGs) of the plant hormone pathway in red (R) and white (W) nut flowers at different development stages. **(A)** Venn diagram of the DEGs related to the plant hormone pathway in four comparison groups. **(B)** Analysis of the DEGs in the different development stages of nut flowers. In the Venn diagram, the *numbers* represent the shared or unique DEG counts in the different comparison groups. In the heat map, the *red* and *blue colors* represent upregulated and downregulated gene expression, respectively.

Plant hormones play an important role in the regulation of plant flowering. Further analysis showed that the DEGs were significantly enriched in the plant hormone signal transduction pathway during the process of nut flower development. Therefore, the expression patterns of the plant hormone regulation-related genes were analyzed in R and W. Of these genes, 205 DEGs in R1 *vs.* R2, 213 DEGs in R2 *vs.* R3, 395 DEGs in W1 *vs.* W2, and 166 DEGs in W2 *vs.* W3 were screened (**Figure 3A**). A total of 137 DEGs were shared in the R1 *vs.* R2 and W1 *vs.* W2 comparison groups. After removing the DEGs whose expression level was less than 10, a total of 77 DEGs were obtained for analysis of the expression patterns (**Figure 3**). The results revealed that, in both R and W, some genes were downregulated while others were upregulated with flower development (**Figure 3B**).

Further analysis showed that the downregulated DEGs included flowering inhibitory genes such as *MYC2* (*Cluster-25626.29606*, *Cluster-25626.151777*, *Cluster-25626.206736*, *Cluster-25626.62227*, and *Cluster-25626.190943*), which inhibit plant flowering through various pathways. In addition, some genes, including *GID1* and *GH3*, were downregulated during flower development. Among them, the upregulated DEGs were mainly related to *ARF* (auxin response factor) (*Cluster-25626.109491*, *Cluster-25626.107280*, and *Cluster-25626.129499*); *IAA* (*Cluster-25626.143080*, *Cluster-25626.100525*, *Cluster-25626.132270*, *Cluster-25626.130747*, and *Cluster-25626.119650*); *SAUR* (*Cluster-25626.164744*, *Cluster-25626.116576*, *Cluster-25626.86188*, *Cluster-25626.106735*, *Cluster-25626.123530*, *Cluster-25626.119538*, *Cluster-25626.127234*, and *Cluster-25626.106483*); and *AUX1* (*Cluster-25626.127778*, *Cluster-25626.81269*, *Cluster-25626.106873*, *Cluster-25626.107669*, *Cluster-25626.107666*, *Cluster-25626.107665*, *Cluster-25626.72889*, and *Cluster-25626.126447*).

In addition, the DEGs related to plant hormone synthesis were involved in *M. integrifolia* flowering in the R2 *vs.* R3 and W2 *vs.* W3 stages. The results showed that these DEGs mainly included 46 common genes. Except for the 13 DEGs shared in the early stage of flower development, there were 33 DEGs in the early stage of flower development (**Figure 3A**). In this study, some genes were significantly enriched in the brassinolide (BR) signal transduction pathway at the later stage of nut flower development, including *BRI1* (*Cluster-25626.145185*, *Cluster-25626.70019*, and *Cluster-25626.45513*); *BAK1* (*Cluster-25626.141544*); and *BSK* (*Cluster-25626.225384* and *Cluster-25626.143495*) (**Figure 3B**). These results indicated that, in the early stage of flower development, most of the DEGs were involved in the IAA signal transduction pathway, which plays a very important role in the early stage of nut flower development (stages 1 and 2). The DEGs in the BR signal transduction pathway play an important role in the late anthesis development of *M. integrifolia* flowers.

### 3.5 Effects of starch and sucrose metabolism on *M. integrifolia* flower development

**Figure 4:**
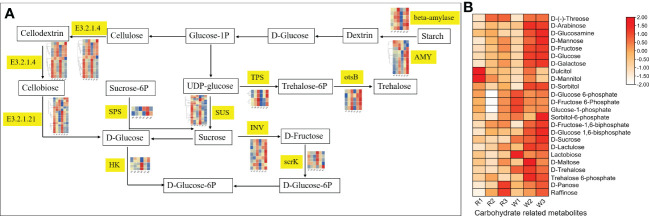
Changes of the gene expression and metabolite accumulation levels of nut flowers in the starch and sucrose metabolism pathway. **(A)** Flow diagram of the starch and sucrose metabolism pathway in different nut flower development groups. The *words in yellow background* indicate the names of the genes involved in the pathway. The small heat map corresponding to the gene name represents the gene expression in the different groups of nut flower. The *red* and *blue colors* in the small heat map indicate upregulated and downregulated gene expression, respectively. **(B)** Accumulation patterns of the carbohydrates involved in the starch and sucrose metabolic pathway in the red (R) and white (W) nut flowers. In the heat map, the *red and blue colors* indicate upregulated and downregulated metabolite accumulation, respectively.

This study also found that the DEGs in nut flowers were significantly enriched in the starch and sucrose metabolism pathways. Therefore, we further analyzed the changes in the gene expression in this pathway (**Figure 4**). Among them, sucrose synthase (SUS), sucrose invertase (INV), and beta-glucosidase (E3.2.1.21) were highly expressed in the early stage of nut flower development, which is consistent with metabolite accumulation. However, the expression of the *TPS* and *ostB* genes was gradually upregulated with the development of flowers (**Figure 4**). These DEGs regulate and hydrolyze the stored starch in nut flower tissue cells for conversion into small molecular sugars, such as sucrose and fructose, for nut flower growth, which also play an important role in the regulation of *M. integrifolia* flowering.

### 3.6 Effects of phenylpropanoid biosynthesis on *M. integrifolia* flower development

**Figure 5:**
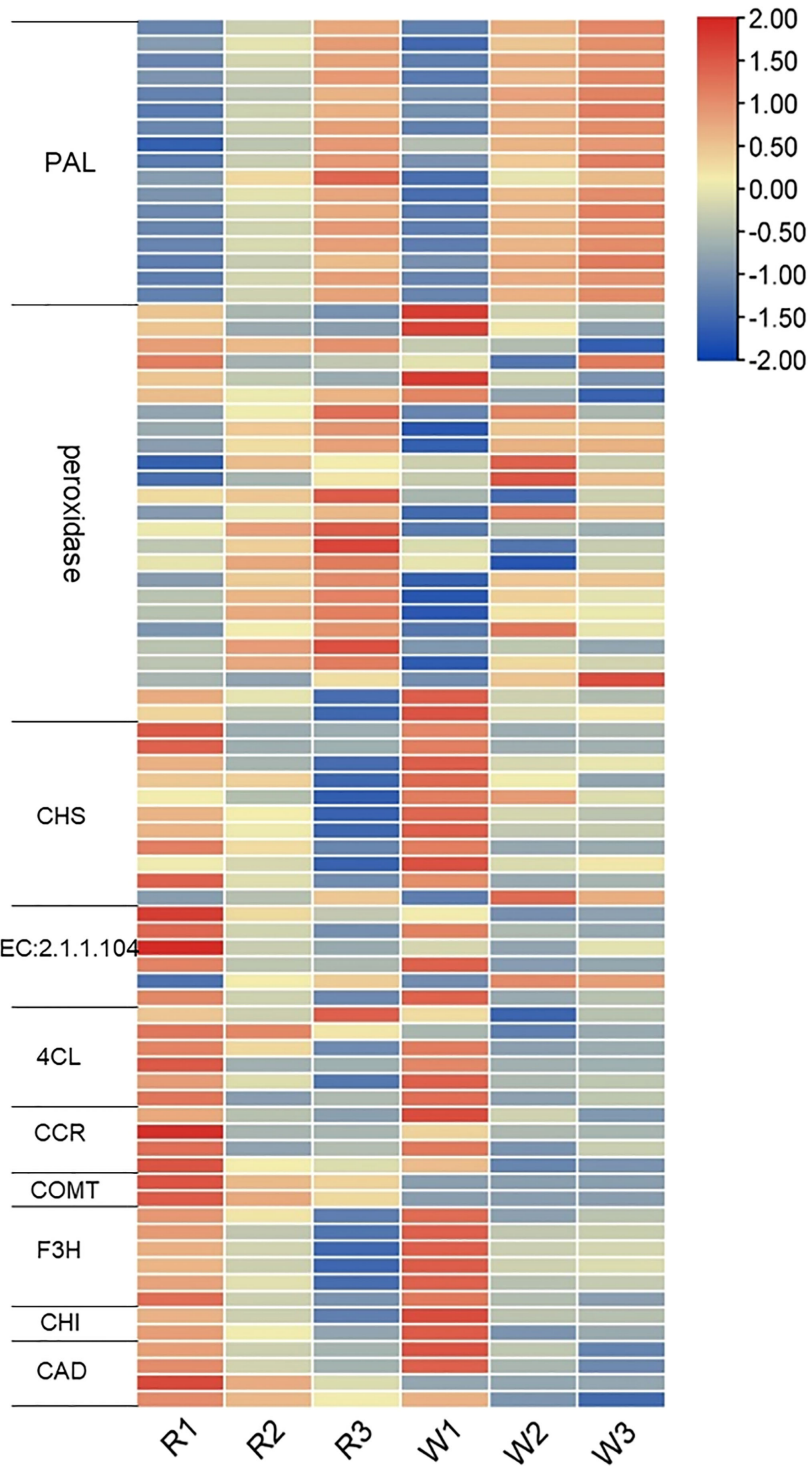
Expression analysis of the differentially expressed genes (DEGs) in the phenylpropane biosynthesis pathway at different nut flower development stages. The *red and blue colors* represent upregulated and downregulated gene expression, respectively. On the *left* are the names and classifications of the genes in the phenylpropane biosynthesis pathway. *PAL*, phenylalanine ammonia-lyase; *CHS*, chalcone synthase; *4CL*, 4-coumarate:CoA ligase; *CCR*, cinnamoyl-CoA reductase gene; *COMT*, catechol-*O*-methyltransferase; *F3H*, flavanone 3-hydroxylase gene; *CHI*, chalcone isomerase gene; *CAD*, cinnamyl-alcohol dehydrogenase.

We analyzed the gene expression changes in phenylpropanoid metabolism, including the phenylpropanoid biosynthesis, flavonoid biosynthesis, anthocyanin biosynthesis, flavonoid and flavonol biosynthesis, and isoflavone biosynthesis pathways. Previously, we have analyzed the relationship between anthocyanin synthesis and nut flower color formation. Therefore, we further analyzed the changes in the gene expression in these metabolic pathways. A total of 83 DEGs were identified in this study (**Figure 5**). The expression level of phenylalanine ammonia-lyase (*PAL*) (upstream of these pathways) was gradually upregulated with the development of nut flowers (**Figure 5**). The results showed that the phenylpropanoid biosynthesis pathway was activated during nut flower development. The downstream key genes (peroxidase) of the lignin biosynthesis pathway also basically showed upregulation, while the key genes of the flavonoid biosynthesis pathway, such as chalcone synthase (*CHS*), chalcone isomerase gene (*CHI*), flavanone 3-hydroxylase gene (*F3H*), and 4-coumarate:CoA ligase (*4CL*), were downregulated. These results suggested that, with the development of nut flowers, the gene and related metabolites in the phenylpropanoid biosynthesis pathway mainly flow to lignin biosynthesis branches, while the flavonoid biosynthesis pathway is weakened. The anthocyanin biosynthesis pathway gene and related metabolite synthesis variation was also consistent with that of the flavonoid biosynthesis pathway. In addition, the activation of the phenylalanine pathway during nut flower development may be related to flower aroma formation. Therefore, we further analyzed the VOCs in each stage of *M. integrifolia* flower development.

### 3.7 Analysis of the aroma in *M. integrifolia* flowers

**Figure 6:**
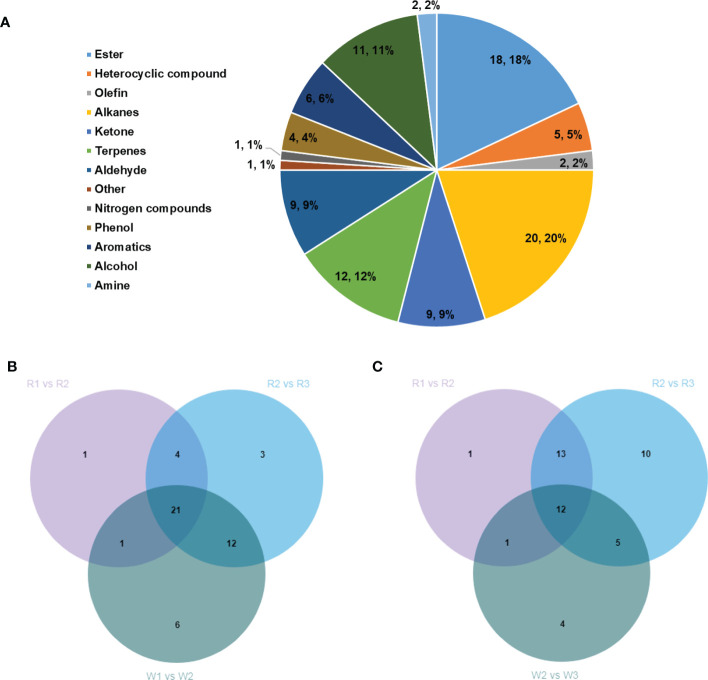
**(A)** Classification and proportion of a total of 100 volatile organic compounds (VOCs) detected in *Macadamia integrifolia* flowers at different development stages. **(B)** Venn diagram analysis of the upregulated VOCs in the W1 *vs.* W2 group compared with the R1 *vs.* R2 and R2 *vs.* R3 nut flower comparison groups. **(C)** Venn diagram analysis of the upregulated VOCs in the W2 *vs.* W3 group compared with the R1 *vs.* R2 and R2 *vs.* R3 nut flower comparison groups. The *percentages* in the pie chart represent the total proportions of the different classified VOCs in nut flowers. The *numbers* in the Venn diagram represent the shared or unique VOC counts in the different comparison groups. *R* and *W* denote red and white nut flowers, respectively.

Only a few studies have revealed the mechanism of aroma formation during nut flower development. Therefore, to elucidate this mechanism, GC-MS and LC-MS were used to detect the VOCs (aromas) in R and W in this study. A total of 100 VOCs were identified, including esters (18), alkanes (20), ketones (9), terpenes (12), aldehydes (9), and alcohols (11) (**Figure 6A**). We analyzed the correlation between the differentially accumulated VOCs and associated DEGs in this study. The VOCs accumulated in the different stages of flower development were screened based on fold change ≥2, fold change ≤0.5, VIP ≥ 1, and *p* < 0.05. The results showed that the contents of VOCs increased gradually with the development of flowers in both R and W (**Supplementary Figure S3**). However, interestingly, the accumulation of VOCs mainly occurred in the later stages of R, with 27 in the R1 *vs.* R2 group and 40 in the R2 *vs.* R3 group, which were significantly upregulated (**Supplementary Figures S3A, B**). Such VOCs may be present in the form of precursor metabolites at the early stage (SA). Therefore, they have not been detected by GC-MS. Our results showed that these aroma-related VOCs were produced only at certain stages during flowering. However, the accumulation of VOCs seemed to occur mainly in the early stage of flower development in W (40 in W1 *vs.* W2 and 20 in W2 *vs.* W3) (**Supplementary Figures S3C, D**). Furthermore, we conducted Venn analysis of the DEGs (upregulated) and found that most of the substances in W1 *vs.* W2 (**Figure 6B**) overlapped with those in R2 *vs.* R3, while only six were unique. Similarly, in W2 *vs.* W3 (**Figure 6C**), there were only four unique differences in the accumulation of VOCs. The results showed that there was no significant difference in the aroma composition between R and W, but the main difference was reflected in the time of aroma formation (flower development stage). The formation of flower VOCs (aroma) in W was earlier than that in R. This may be the main factor leading to the difference in yield between the two nut varieties.

### 3.8 Combined transcriptome and metabolome analysis

**Figure 7:**
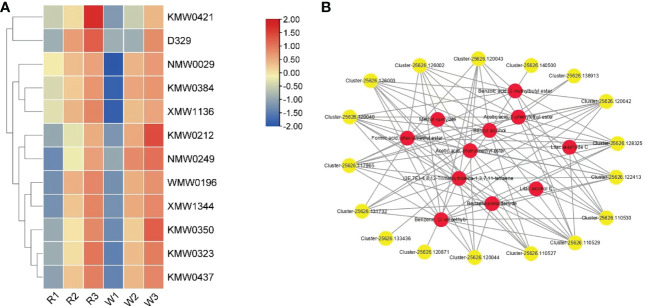
**(A)** Accumulation patterns of the 12 volatile organic compounds (VOCs) in *Macadamia integrifolia* flowers at the R1, R2, R3, W1, W2, and W3 stages (R and W refer to red and white nut flowers, respectively). The *red* and *blue colors* represent up-accumulated and down-accumulated VOCs, respectively. **(B)** STRING network analysis of the differentially expressed genes (DEGs) with shared differentially accumulated VOCs in nut flowers. The correlation in each gene and related VOCs was greater than 0.8. In the network diagram, *yellow dots* represent the key genes and *red dots* represent the associated VOC metabolites. The *solid lines* and *line numbers* indicate the strength of the correlation between the genes and metabolites.

Ultimately, 12 different accumulative VOCs were screened out at different stages of *M. integrifolia* flower development (**Table 3**). These differentially accumulated VOCs were critical for the aroma formation of *M. integrifolia* flowers. The contents of these VOCs accumulated gradually with the development of flowers. In addition, the accumulation of VOCs in W was significantly higher than that in R. It was speculated that these metabolites are extremely important for the formation of the flower aroma of *M. integrifolia* (**Figure 7A**). Therefore, the results showed that the synthesis of VOCs in W was earlier than that in R, which is consistent with the phenotypic results of the two varieties. In this experiment, 17 genes were identified in the combined analysis of the metabolome and transcriptome, which were significantly associated with 12 DAMs, and their contents were regulated by the expression of these genes. These genes included three *AAT* genes, one *LOX* gene, and 13 *PAL* genes (**Figure 7B**). PAL is a key enzyme of the phenylpropanoid pathway that catalyzes the deamination of phenylalanine to *trans*-cinnamic acid, a precursor for the lignin and flavonoid biosynthetic pathways. To date, *PAL* genes have been less extensively studied in gymnosperms than in angiosperms. The key DEGs involved in DAM synthesis were verified by qRT-PCR. The results suggested that these genes are related to the synthesis of key metabolites during *M. integrifolia* flower development, thus regulating the development of the different varieties of flowers. Therefore, in this experiment, these key DEGs were used as the candidate genes for further functional verification.

**Table 3:** Twelve different accumulative volatile organic compounds (VOCs) at different stages of *Macadamia integrifolia* flower development.

Index	Compounds	Exact mass (Da)	Formula	NIST_RI	Class	CAS	RT	RI	Match factor
KMW0323	Benzyl alcohol	1.08E^+02^	C_7_H_8_O	1.04E^+03^	Alcohol	100-51-6	1.20E^+01^	1.03E^+03^	9.84E^+01^
KMW0384	(2-Nitroethyl)benzene	1.51E^+02^	C_8_H_9_NO_2_	1.19E^+03^	Aromatics	6125-24-2	1.97E^+01^	1.30E^+03^	9.73E^+01^
KMW0212	Benzeneacetaldehyde	1.20E^+02^	C_8_H_8_O	1.05E^+03^	Aldehyde	122-78-1	1.22E^+01^	1.04E^+03^	9.56E^+01^
KMW0350	Acetic acid, phenylmethyl ester	1.50E^+02^	C_9_H_10_O_2_	1.16E^+03^	Ester	140-11-4	1.58E^+01^	1.16E^+03^	7.82E^+01^
KMW0421	Methyl salicylate	1.52E^+02^	C_8_H_8_O_3_	1.23E^+03^	Ester	119-36-8	1.67E^+01^	1.19E^+03^	7.92E^+01^
KMW0437	Acetic acid, 2-phenylethyl ester	1.64E^+02^	C_10_H_12_O_2_	1.26E^+03^	Ester	103-45-7	1.84E^+01^	1.25E^+03^	9.71E^+01^
WMW0196	Lilac aldehyde C	1.68E^+02^	C_10_H_16_O_2_	1.20E^+03^	Aldehyde	53447-48-6	1.52E^+01^	1.14E^+03^	9.03E^+01^
NMW0029	Formic acid, phenylmethyl ester	1.36E^+02^	C_8_H_8_O_2_	1.08E^+03^	Ester	104-57-4	1.32E^+01^	1.08E^+03^	9.00E^+01^
NMW0249	(3E,7E)-4,8,12-Trimethyltrideca-1,3,7, 11-tetraene	2.18E^+02^	C_16_H_26_	1.58E^+03^	Terpenes	62235-06-7	2.67E^+01^	1.57E^+03^	9.53E^+01^
XMW1136	Lilac alcohol C	1.70E^+02^	C_10_H_18_O_2_	1.22E^+03^	Alcohol	33081-36-6	1.72E^+01^	1.21E^+03^	9.40E^+01^
XMW1344	Benzoic acid, 2-methylbutyl ester	1.92E^+02^	C_12_H_16_O_2_	1.39E^+03^	Ester	1000367-91-3	2.34E^+01^	1.44E^+03^	9.21E^+01^
D329	Myroxide	1.52E^+02^	C_10_H_16_O	1.14E^+03^	Terpenes	28977-57-3	1.89E^+01^	1.27E^+03^	6.61E^+01^

## 4 Discussion

Global crop yields are currently trending below the anticipated food demand (Ray et al., 2013; Fróna et al., 2019). Tree crops contribute over 600 million tons of the 10,600 million tons of annual global food production, and fruit number and fruit size are the key components of tree crop yield (Nyomora et al., 1999; Garner et al., 2011; Patrick and Colyvas, 2014). Macadamia (*M. integrifolia*, *Macadamia tetraphylla*, and hybrids) is a subtropical nut crop that produces up to 3,500 racemes per tree annually (Moncur et al., 1985; McFadyen et al., 2011; Olesen et al., 2011). Each raceme has between 100 and 300 flowers (Trueman et al., 2022). Low and inconsistent macadamia yields are often attributed to low levels of initial fruit set, poor fruit retention, and variations in the nut and kernel size (Trueman et al., 1994; Howlett et al., 2019; Kämper et al., 2019; Trueman et al., 2022). Macadamia has a very low fruit-to-flower ratio, which is common among species of the Proteaceae family (Stephenson, 1981; Ayre and Whelan, 1989). Typically, less than 2% of macadamia flowers develop into mature fruits. The fruit setting rate, yield, and quality of the nuts are closely related to the pollination and development of nut flowers. Therefore, we carried out research on nut flowers in order to contribute to the development of the yield and quality of nuts.

With the gradual improvements in the study of flower aroma metabolic engineering, aroma-related substances can be identified in more plants, aroma synthesis pathways can be more clearly elucidated, and more related enzyme genes can be cloned in the future. However, due to the complexity of the flower aroma metabolic pathway and the specificity of plant species and genera, there are still a lot of problems in genetic engineering related to the flower aroma metabolic pathway (Jin et al., 2020). For example, the terpenoid metabolic pathway produces not only a large number of substances related to flower aroma but also some substances related to plant physiological activities, such as abscisic acid and ethylene, and sometimes some metabolic disorders (Jin et al., 2020). In addition, there are still many gaps in the study of phenylpropanes and fatty acids. Further studies are needed to determine the biosynthesis-related genes of phenylpropanes and aliphatic groups and how these genes are regulated by transcription factors. The relationship between nut flower development and flower color formation was investigated from four perspectives: plant hormone signal transduction, starch and sucrose and phenylpropane metabolism, flavonoid biosynthesis pathway, and anthocyanin biosynthesis pathway.

Macadamia flowers are protandrous and have bisexual flowers that are partially self-incompatible (Sedgley, 1983; Trueman, 2013), but the pollen movement between genotypes (cultivars) has positive effects on the yield, improving both nut retention and maximal nut weight (Howlett et al., 2019; Langdon et al., 2020). Dependence on cross-pollination varies between cultivars (Langdon et al., 2019). Many cultivars predominantly set cross-pollinated nuts, even when cross-pollen donors are not interplanted within the block (Richards et al., 2020). Honey bees (*Apis mellifera*) and stingless bees (*Tetragonula* spp., predominantly *Tetragonula carbonaria*) are pollen foraging workers that are effective pollinators of macadamia (Heard and Exley, 1994; Rhodes, 2001; Evansl et al., 2021). The VOCs (aroma) of flowers comprise the main mechanism to attracting bees and other insects to pollinate, thus affecting the fruit setting rate and the yield of nuts. In this study, 10 different types of VOCs and 12 different types of DAMs were found in the macadamia R and W nut flower varieties. The results of the present study suggested that the R nut variety has a large number of flowers and blooms later every year, which can better attract stingless bees for pollination. At the same time, the accumulation of VOCs mainly occurred in the later stage (40 metabolites were significantly accumulated) in R. However, in W, the accumulation of VOCs mainly occurred in the early stage of nut flower development. The period of aroma formation may be related to the attraction of stingless bees. These key metabolites and VOC synthesis were regulated by three *AAT* genes, one *LOX* gene, and 13 *PAL* genes during nut flower development, thus regulating the development of the different varieties of nut flowers. Our results may help to clarify the molecular mechanism of the effect of *M. integrifolia* flower development on fruit yield.

Flower aroma consists of a series of low-molecular-weight and volatile compounds. The composition and the contents of flower fragrance volatiles differ in different types of plants. According to the synthetic metabolic pathway, flower aroma volatiles can be divided into three categories: terpenes, phenylpropane compounds, and aliphatic compounds (Dudareva and Pichersky, 2000). Similarly, this study also detected the differential accumulation of these volatile metabolites in the different developmental stages of nut flowers. We focused on the synthetic pathway of phenylpropane compounds. The expression of the *PAL* genes was upregulated, while the key genes of the flavonoid biosynthesis pathway, such as *CHS*, *CHI*, *F3H*, and *4CL*, were downregulated. PAL, 4-hydroxycinnamic acid-4-hydroxylase (C4H), and 4-coumaryl-CoA-ligase (4CL) are three key enzymes in the metabolic pathway of phenylpropane compounds (Wang and Cui, 2009). PAL is the key and the rate-limiting enzyme in the phenylpropanoid metabolic pathway. It catalyzes the first step of the phenylalanine pathway and can catalyze the deamination of phenylalanine to cinnamic acid (CA). CA produces 4-coumarate under the hydroxylation of C4H. C4H is the second step of the phenylalanine pathway that requires the joint action of NADPH and oxygen. It is highly specific to the substrate and is closely related to plant lignin (Li et al., 2007; Liang et al., 2014). On the other hand, 4CL catalyzes the synthesis of CoA esters, such as coumaric acid and CA. These are then further transformed into secondary metabolites such as lignin and flavonoids, which comprise the last step of the phenylalanine pathway and the branching point of the different products formed by phenylpropane metabolism.

Flavonoids are a large group of plant-derived compounds that share a common three-ring phenyl benzopyrone structure and are present in nature as free aglycones or glycosides (Khan et al., 2021). They can be classified into anthocyanins, flavans, flavones, flavanols, flavonols, flavanones, flavonones, and isoflavones, among others, based on the degree of oxidation of the middle pyrone ring or the substitution patterns (Di Carlo et al., 1999). In this study, anthocyanins were identified as the key flavonoids, and four possible nut flower coloring substances were identified: delphindin-3-*O*-glucoside (Pme1398), petunidin-3-*O*-arabinoside (Smlp002277), delphinidin-3-*O*-arabinoside (Smlp001915), and cyanide-3-*O*-arabinoside (Smlp002532). These flavonoids may be involved in the coloration of nut flowers and the formation of floral scent VOCs downstream, which help attract pollinators and increase the fruit setting rate and yield.

In conclusion, the transcriptome and metabolome analyses provided in-depth insights into the dynamic flower coloration of *M. integrifolia*. The results of this study provide a reference for researchers to further study nut flower development, flower aroma, and flower color and also provide insights into improving nut yield. The RNA-seq data from this study will be an essential resource for the further functional study of several traits with genome editing and also for molecular marker-assisted breeding to promote genetic studies and novel cultivar breeding for *M. integrifolia*.

## Data availability statement

The data present in the study are deposited in the NCBI BioProject repository, accession number PRJNA899604.

## Author contributions

YW: Literature search, analysis, investigation, resources, and writing—original draft. JX: Investigation, metabolite analysis, methodology, and writing—review and editing. ZW: Resources, bioinformatic analysis, and writing—review and editing. ZY: Investigation, and sample collection. ZX: Investigation and transcriptome analysis. CW and RS: Conceptualization, experimental design, obtaining research funds, resources, supervision, and writing—review and editing. All authors contributed to the article and approved the submitted version.
