# Supplementary material for: Combined analysis of multi-omics reveals the potential mechanism of flower color and aroma formation in Macadamia integrifolia
Source: Front Plant Sci. 2023 Feb 1;13:1095644. doi: 10.3389/fpls.2022.1095644 (PMC9931397; doi:10.3389/fpls.2022.1095644)
Supplement: Supplementary file 1 [file DataSheet_1.docx]

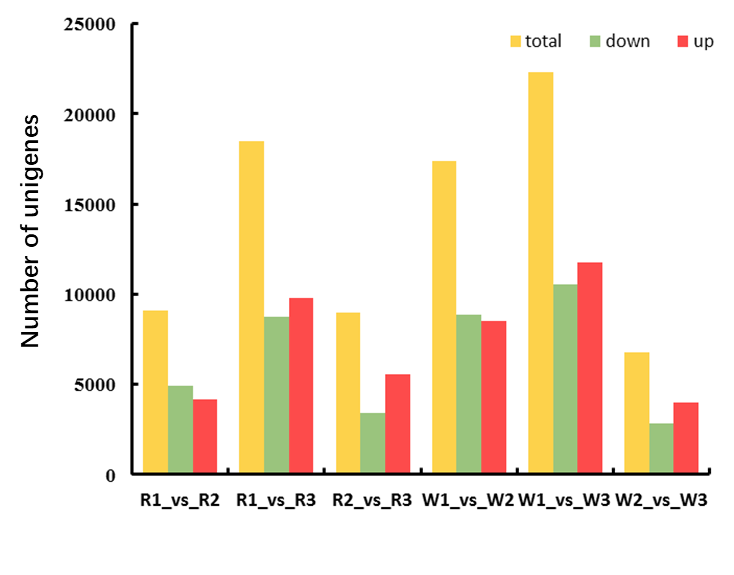


Fig. S1 Statistics of the number of DEGs among different comparison groups of *M. integrifolia* flowers. (The yellow column shows the total number of DEGs, the green column represents the down-regulated DEGs, and the red column represents the up-regulated DEGs.)


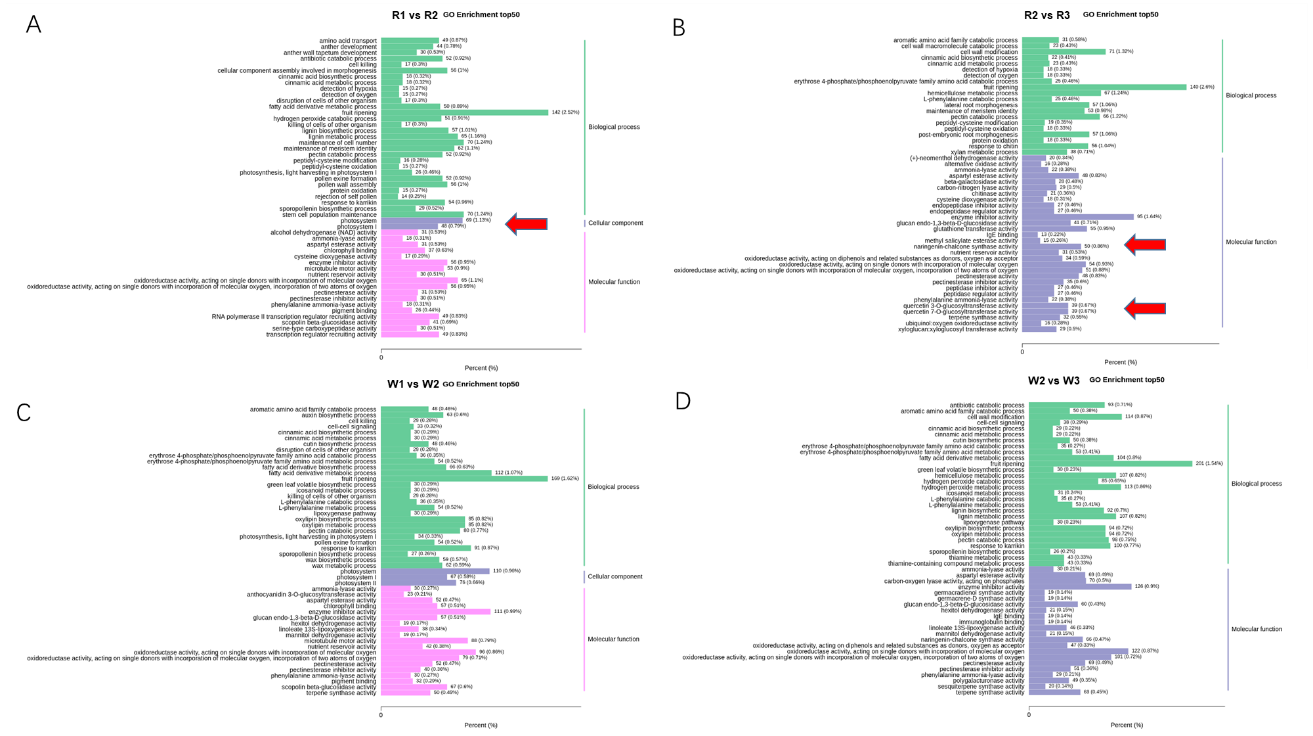


Fig. S2 Top50 of GO enrichment items of DEGs in *M. integrifolia* flowers at different developmental stages. R1 vs R2 (A), R2 vs R3 (B), W1 vs W2 (C) and W2 vs W3 (D). In the graphic, the green color terms represent biological process, the purple color terms represent cellular component, and the pink color terms represent molecular function.


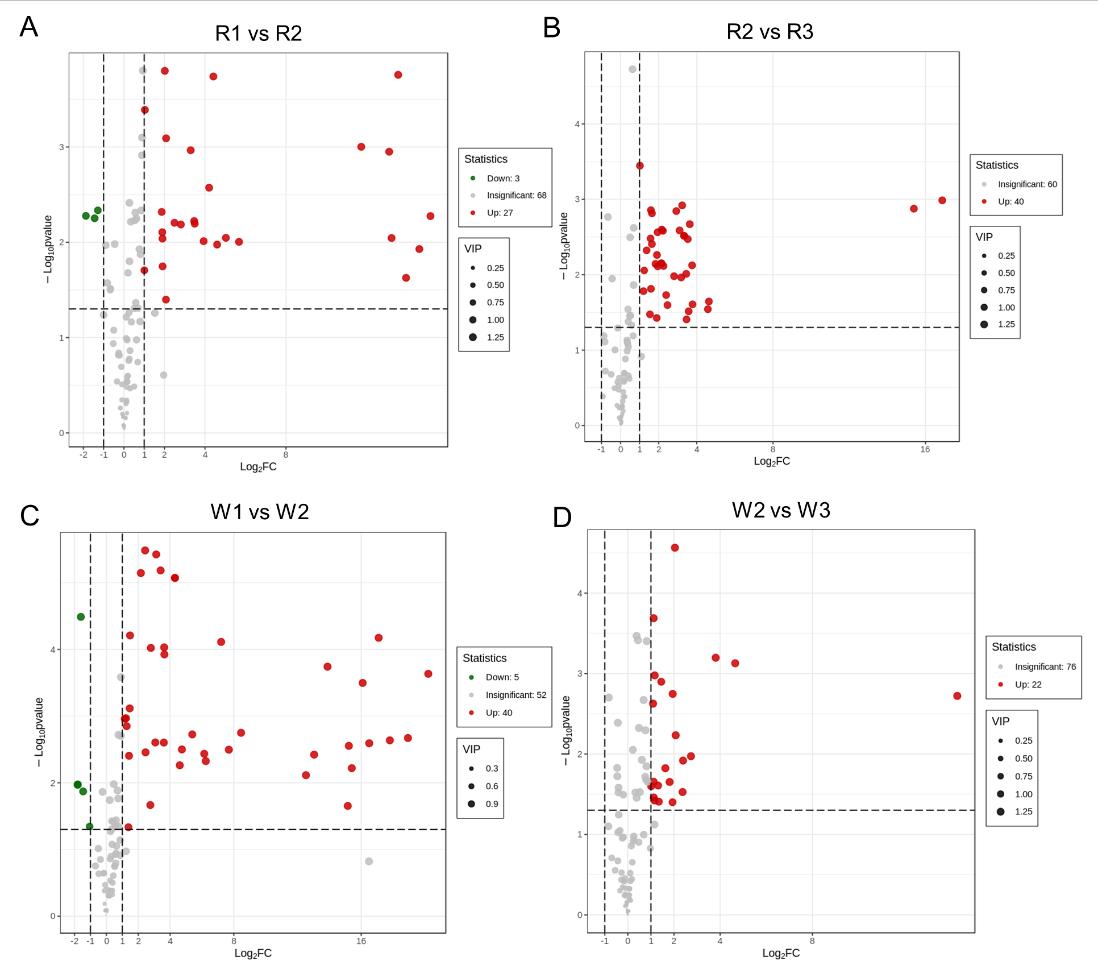


Fig. S3 Volcano plot of the accumulation of different volatile organic compounds (VOCs). The X coordinate was |log2(fold change) | and the Y coordinate was -log 10 (P value). Each dot represented a gene. Red dots were the up-regulated VOCs of significant accumulation. Green dots were down-regulated VOCs of significant accumulation. Black dots were VOCs of non-significant difference.
